# Real-World Early and Short-Term Outcomes of ERCP-Guided Biliary Stenting in Suspected Malignant or Indeterminate Biliary Strictures

**DOI:** 10.3390/medicina62071276

**Published:** 2026-07-02

**Authors:** Serkan Ademoğlu, Ferudun Kaya

**Affiliations:** Department of Gastroenterologic Surgery, Ministry of Health, Gaziantep City Hospital, 27470 Gaziantep, Türkiye

**Keywords:** ERCP, biliary stenting, suspected malignant biliary stricture, indeterminate biliary stricture, malignant biliary obstruction, stent dysfunction, post-ERCP pancreatitis, clinical drainage success

## Abstract

*Background and Objectives*: Biliary strictures considered malignant or indeterminate at the time of endoscopic retrograde cholangiopancreatography (ERCP) frequently require endoscopic biliary drainage for biochemical improvement, symptom control, and continuation of diagnostic or oncologic management. This study aimed to evaluate the early and short-term real-world outcomes of ERCP-guided biliary stenting in patients with biliary strictures considered malignant or indeterminate at the time of ERCP, including cases in which malignancy was not subsequently confirmed. *Materials and Methods*: This single-center retrospective observational study screened 996 analyzable ERCP records performed between 27 February 2024 and 27 April 2026. The final cohort included 164 ERCP-guided biliary stenting procedures performed in 162 patients with suspected malignant or indeterminate biliary strictures. Clinical drainage success was defined according to the treating endoscopist’s documented assessment based on clinical improvement and/or biochemical bilirubin decline after stenting. *Results*: The median age was 60.0 years, and 87 procedures (53.0%) were performed in female patients. Definite malignancy was documented in 121 cases (73.8%). Distal strictures were the most common localization (72.0%). Clinical drainage success was achieved in 153 cases (93.3%). Median total bilirubin decreased from 1.50 mg/dL before ERCP to 0.42 mg/dL on post-ERCP day 14 (*p* < 0.001). ERCP-related adverse events occurred in 18 cases (11.0%), including post-ERCP pancreatitis in 10 cases (6.1%). Thirty-day mortality occurred in 2 cases (1.2%). Stent dysfunction and repeat ERCP were each observed in 23 cases (14.0%). Using a pre-ERCP total bilirubin threshold of >3.0 mg/dL, jaundice at presentation was present in 61 procedures (37.2%). Clinical drainage success was 91.8% in jaundiced procedures and 94.2% in non-jaundiced procedures. In a restricted multivariable model including only ASA physical status ≥3 and stent type, ASA physical status ≥3 showed an exploratory association with stent dysfunction (adjusted odds ratio: 4.84; 95% confidence interval: 1.36–17.23; *p* = 0.015). Although adverse event rates differed between plastic and metal stent groups, these comparisons were limited by baseline imbalance. *Conclusions*: ERCP-guided biliary stenting provided high clinical drainage success and significant early bilirubin reduction in patients with suspected malignant or indeterminate biliary strictures. Subgroup analyses suggested that stricture localization influenced real-world stent selection, whereas clinical drainage success and stent dysfunction did not differ significantly between distal and perihilar/hilar strictures. Stent dysfunction and repeat ERCP were required in a minority of cases, and higher ASA physical status showed an exploratory association with stent dysfunction, but this finding should be interpreted cautiously because of the limited number of dysfunction events. Stent-type comparisons should be interpreted cautiously because of real-world selection bias.

## 1. Introduction

Malignant biliary obstruction is a clinically important condition encountered in patients with pancreatobiliary and periampullary malignancies. Pancreatic cancer and biliary tract cancers remain major causes of cancer-related morbidity and mortality, and biliary obstruction may complicate the diagnostic, surgical, and oncologic management of these patients [1]. In daily endoscopic practice, malignant biliary obstruction may involve distal, perihilar, or hilar bile ducts and may present with jaundice, cholangitis, pruritus, impaired liver function, or the need for biliary decompression before further treatment [2].

The evaluation of biliary strictures requires both diagnostic clarification and therapeutic planning. Current clinical guidelines emphasize that the diagnostic approach should be individualized according to stricture location, clinical presentation, the presence of a pancreatic or abdominal mass, and the feasibility of tissue acquisition [3,4]. In patients with suspected or indeterminate malignant biliary strictures, endoscopic retrograde cholangiopancreatography (ERCP)-based sampling, brush cytology, fluoroscopy-guided biopsy, cholangioscopy, and endoscopic ultrasound-guided tissue acquisition may be used according to the clinical scenario and available expertise [5].

Biliary drainage is a central component of management when obstruction is clinically relevant. The optimal drainage strategy differs between distal and hilar obstruction because of anatomical complexity, drainage targets, resectability status, expected treatment pathway, and anticipated duration of stent patency [6]. ERCP-guided transpapillary biliary stenting remains one of the main approaches for biliary decompression, and stent selection is generally influenced by stricture location, diagnostic certainty, suspected or confirmed malignancy, and whether drainage is performed for palliation or as a bridge to surgery or systemic treatment [7,8].

In suspected malignant biliary strictures, real-world decision-making is often more heterogeneous than guideline algorithms. At the time of index ERCP, malignancy may be suspected but not yet pathologically confirmed, and treatment decisions may need to be made before the diagnostic process is fully completed. This is particularly relevant in patients with obstructive jaundice, cholangitis, impaired clinical condition, or planned oncologic treatment. Contemporary oncologic guidelines for biliary tract and pancreatic cancers emphasize multidisciplinary management, appropriate staging, tissue diagnosis when indicated, selective biliary drainage, and timely transition to surgical or systemic treatment pathways [9,10].

Therefore, real-world data on ERCP-guided biliary stenting in suspected malignant biliary strictures may provide clinically useful information beyond technical success alone. Outcomes such as clinical drainage success, bilirubin response, ERCP-related adverse events, stent dysfunction, repeat ERCP requirement, and early mortality are directly relevant to patient management. The present study aimed to evaluate the early and short-term real-world outcomes of ERCP-guided biliary stenting in patients with biliary strictures considered malignant or indeterminate at the time of ERCP. Specifically, we assessed clinical drainage success, bilirubin response, adverse events, stent dysfunction, repeat ERCP requirement, and factors associated with stent dysfunction in a single-center retrospective cohort.

## 2. Materials and Methods

### 2.1. Study Design and Ethical Approval

This single-center, retrospective observational study was conducted at the Endoscopy Unit of Gaziantep City Hospital, Türkiye. This single-center, retrospective observational study evaluated real-world early and short-term outcomes of endoscopic retrograde cholangiopancreatography (ERCP)-guided biliary stenting in patients with suspected malignant or indeterminate biliary strictures.

The study was approved by the Gaziantep City Hospital Non-Interventional Clinical Research Ethics Committee (decision no: 449/2026; approval date: 18 February 2026). All data were retrospectively collected from hospital records and anonymized before analysis. No additional intervention, laboratory test, imaging procedure, or follow-up visit was performed for study purposes.

### 2.2. Study Population

All ERCP records dated between 27 February 2024 and 27 April 2026 were retrospectively screened. After exclusion of records with missing essential procedural information, 996 analyzable ERCP records constituted the screened population.

The final study cohort included ERCP procedures that fulfilled all of the following criteria:

1. Suspected malignant biliary obstruction based on clinical, radiological, endoscopic, pathological, or oncological records;
2. Presence of biliary stricture documented during the diagnostic or therapeutic evaluation;
3. Placement of a biliary stent during ERCP.

The term “suspected malignant or indeterminate biliary stricture” was used to reflect the clinical status at the time of ERCP rather than a requirement for subsequent pathological confirmation. Therefore, patients were not excluded solely because malignancy was not definitively documented during follow-up, provided that the index ERCP was performed for a stricture considered malignant or indeterminate in the clinical, radiological, endoscopic, pathological, or oncological records.

Accordingly, 164 ERCP-guided biliary stenting procedures performed in 162 patients were included in the final analysis. Procedures performed for non-stricture indications, such as isolated choledocholithiasis without suspected malignant biliary stricture, and procedures in which biliary stenting was not performed were excluded from the final cohort.

The primary unit of analysis was the eligible ERCP-guided biliary stenting procedure. Because two patients had more than one eligible procedure during the study period, both the number of procedures and the number of individual patients were reported.

### 2.3. Data Collection and Variables

Data were retrospectively extracted from electronic hospital records, laboratory databases, imaging reports, pathology records, oncological records, and ERCP procedure reports.

The following variables were recorded: age, sex, hospitalization status, type of presentation, comorbidities, ASA score, ERCP indication, baseline laboratory values, pre-ERCP total bilirubin, post-ERCP day 14 total bilirubin, ERCP date, last follow-up date, sedation type, papilla characteristics, cannulation success, sphincterotomy, pre-cut sphincterotomy, common bile duct diameter, presence of stones, stent placement, stent type, number of stents, stricture localization, suspected malignancy, definite malignancy diagnosis, biopsy or brush cytology, pathology result, clinical drainage success, ERCP-related adverse events, post-ERCP pancreatitis, bleeding, perforation, cholangitis, ICU requirement, 30-day mortality, stent dysfunction, type of stent dysfunction, repeat ERCP, reason for repeat ERCP, time to stent dysfunction, hospital length of stay, and follow-up duration.

Stricture localization was categorized as distal, perihilar, or hilar according to ERCP findings and available radiological or endoscopic documentation. Stent type was categorized as plastic stent or metal stent. Definite malignancy was recorded when malignancy was documented in pathology reports or clinical oncological records.

### 2.4. Definitions and Outcomes

The primary outcomes were clinical drainage success, bilirubin response, ERCP-related adverse events, stent dysfunction, and repeat ERCP requirement.

Clinical drainage success was defined as successful biliary stent placement across the target stricture with documented clinical and/or biochemical improvement during early follow-up, without the need for immediate alternative biliary drainage. Jaundice at presentation was operationally defined as a pre-ERCP total bilirubin level >3.0 mg/dL. In patients without jaundice, biliary stricture was accepted only when a stricture was documented in radiological, endoscopic, pathological, oncological, or ERCP records. In these cases, stent placement was performed for a clinically relevant suspected malignant or indeterminate biliary stricture, including documented ductal narrowing, upstream biliary dilatation, cholestatic biochemical abnormalities, cholangitis, suspected malignant compression, planned oncological or surgical management, or the need for temporary drainage during diagnostic clarification. Therefore, clinical drainage success in non-jaundiced patients was not assessed solely by bilirubin decline, but by successful stent deployment across the target stricture, documented clinical and/or biochemical stabilization or improvement, and absence of immediate alternative biliary drainage requirement. Bilirubin response was evaluated by comparing pre-ERCP total bilirubin levels with total bilirubin levels measured on post-ERCP day 14. The day-14 bilirubin value represented the recorded laboratory value closest to post-ERCP day 14 in routine clinical follow-up and was not imputed or truncated for statistical purposes. No bilirubin value was imputed for the analysis. Pre-ERCP and post-ERCP day-14 total bilirubin values were extracted as recorded in the hospital laboratory database. When identical low-normal values were repeatedly recorded, these values were retained as raw laboratory values and were not generated by statistical truncation or missing-data handling. For clarity, non-jaundiced procedures did not represent prophylactic or incidental stenting; in all cases, ERCP-guided stenting was performed because a clinically relevant biliary stricture was documented or strongly suspected in the clinical context, and drainage was considered necessary for symptom control, cholangitis or cholestatic abnormalities, relief of radiological obstruction, or continuation of diagnostic, oncological, or surgical management.

Stent dysfunction was defined as documented stent occlusion, migration, malposition, insufficient drainage, or recurrent biliary obstruction requiring clinical reassessment and/or repeat ERCP. Time to stent dysfunction was calculated from the index ERCP date to the date of documented stent dysfunction and was analyzed only among cases with stent dysfunction.

Post-procedural cholangitis was evaluated according to the diagnostic framework of the Tokyo Guidelines 2018 for acute cholangitis [11,12]. ERCP-related adverse events and their severity were classified using established endoscopic adverse event terminology and ERCP complication consensus documents [13,14,15]. Post-ERCP pancreatitis was defined according to standard consensus criteria and contemporary guideline recommendations [14,16]. In patients without contraindications, pharmacological prophylaxis for post-ERCP pancreatitis was administered according to routine clinical practice, including rectal nonsteroidal anti-inflammatory drugs. Prophylactic pancreatic duct stenting was performed selectively in high-risk cases at the discretion of the endoscopist.

Secondary outcomes included ICU requirement, 30-day mortality, hospital length of stay, follow-up duration, and factors associated with stent dysfunction. Thirty-day mortality was defined as all-cause mortality occurring within 30 days after the index ERCP procedure.

### 2.5. Statistical Analysis

Data cleaning, consistency checks, and verification of derived variables were performed using Microsoft Excel for Microsoft 365 (Microsoft Corp., Redmond, WA, USA) and Python version 3.12 (Python Software Foundation, Wilmington, DE, USA)-based data management tools. Final statistical analyses were performed using IBM SPSS Statistics for Windows, Version 26.0 (IBM Corp., Armonk, NY, USA).

Continuous variables were evaluated for distributional characteristics using visual inspection and normality testing. Since most continuous variables were not normally distributed, they were presented as median and interquartile range. Categorical variables were presented as number and percentage. Because bilirubin values were markedly skewed, median and interquartile range were used as the primary descriptive statistics; however, mean ± standard deviation and range were additionally reported for pre-ERCP and post-ERCP day-14 total bilirubin levels to improve interpretability. For paired comparisons of pre-ERCP and post-ERCP day-14 total bilirubin levels, the Wilcoxon signed-rank test was used. For comparisons between two independent groups, the Mann–Whitney U test was used for continuous variables, and the chi-square test or Fisher’s exact test was used for categorical variables, as appropriate. Logistic regression analysis was performed to identify factors associated with stent dysfunction. Univariable logistic regression was first conducted for clinically relevant variables. Given the limited number of stent dysfunction events, multivariable modeling was intentionally restricted to reduce the risk of overfitting, and a restricted adjusted model including ASA score ≥ 3 and stent type was constructed. Results were reported as odds ratios with 95% confidence intervals. Stent dysfunction-free probability was evaluated exploratorily using the Kaplan–Meier method and compared between plastic and metal stent groups using the log-rank test. The event was defined as the first documented stent dysfunction after the index ERCP. Procedures without documented stent dysfunction were censored at the last available follow-up. Death or absence of further biliary follow-up without documented stent dysfunction was treated as censoring rather than as a competing event. Because follow-up duration differed substantially between stent groups, time-to-event comparisons were interpreted descriptively and not as evidence of comparative stent effectiveness. Missing data were handled by pairwise exclusion for the relevant analysis. All statistical tests were two-sided, and a *p*-value < 0.05 was considered statistically significant.

## 3. Results

### 3.1. Study Selection and Baseline Characteristics

**Figure 1:**
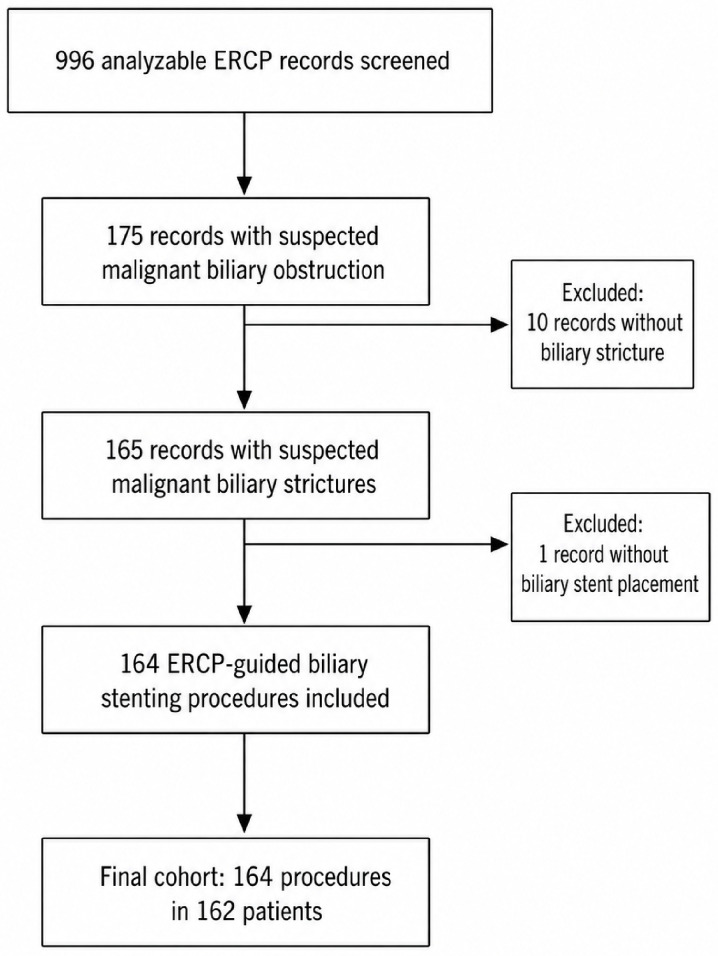
Study flow diagram.

A total of 996 analyzable ERCP records were retrospectively screened during the study period. Among these, 175 records were identified as having suspected malignant biliary obstruction. After exclusion of 10 records without biliary stricture and 1 record without biliary stent placement, 164 ERCP-guided biliary stenting procedures performed in 162 patients were included in the final analysis (Figure 1).

The median age was 60.0 years (IQR, 42.5–70.3), and 87 procedures (53.0%) were performed in female patients. Most cases had an ASA score ≥ 3 (104/164, 63.4%). A definite malignancy diagnosis was present in 121 cases (73.8%).

No definite malignancy was documented during follow-up in 43 procedures (26.2%). These cases were retained in the study cohort because the inclusion criterion was suspected malignant biliary stricture at the time of ERCP rather than histologically confirmed malignancy. In this subgroup, final clinical documentation most frequently indicated stone-related or indeterminate benign biliary obstruction with stricture, and oncological follow-up or palliation was not recorded.

Distal biliary strictures were the most common localization (118/164, 72.0%), followed by perihilar (25/164, 15.2%) and hilar strictures (21/164, 12.8%). Plastic stents were used in 109 cases (66.5%), while metal stents were used in 55 cases (33.5%). Baseline and procedural characteristics of the study cohort are summarized in Table 1.

Using a pre-ERCP total bilirubin threshold of >3.0 mg/dL, jaundice at presentation was present in 61 procedures (37.2%), whereas 103 procedures (62.8%) were classified as non-jaundiced. Among non-jaundiced procedures, definite malignancy was documented in 80 cases (77.7%), distal stricture localization was present in 79 cases (76.7%), and clinical drainage success was achieved in 97 cases (94.2%). Clinical drainage success was 56 of 61 procedures (91.8%) in jaundiced patients and 97 of 103 procedures (94.2%) in non-jaundiced patients. In the non-jaundiced subgroup, the indication for stenting was based on a clinically relevant suspected malignant or indeterminate biliary stricture rather than on hyperbilirubinemia alone. These procedures included patients with documented biliary narrowing and/or upstream biliary dilatation, cholestatic enzyme abnormalities, cholangitis, suspected malignant extrinsic compression, or the need to secure biliary drainage during ongoing diagnostic, oncological, or surgical planning. Therefore, clinical drainage success in this subgroup was assessed by successful stent deployment across the target stricture, documented clinical and/or biochemical stabilization or improvement, and absence of immediate alternative biliary drainage requirement, rather than by bilirubin reduction alone.

Successful biliary stent deployment was an inclusion criterion and was confirmed in all included procedures. The apparent difference between cannulation success and stent deployment success resulted from retrospective data-field availability: the cannulation-status field was documented in 163 of 164 procedures and was not used as the denominator for final stent deployment.

The lower recorded cannulation success rate compared with successful biliary stent deployment reflected incomplete retrospective documentation of the cannulation-status field in one procedure rather than failure of final stent deployment.

### 3.2. Bilirubin Response After ERCP-Guided Stenting

**Figure 2:**
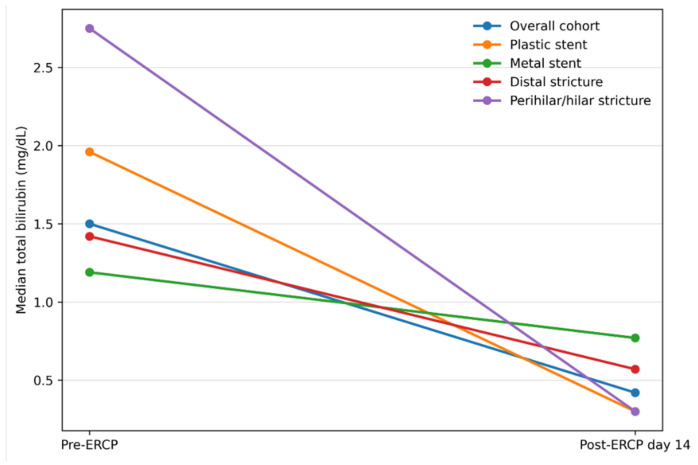
Total bilirubin response after ERCP-guided biliary stenting.

The median pre-ERCP total bilirubin level decreased from 1.50 mg/dL (IQR, 0.80–5.93) to 0.42 mg/dL (IQR, 0.30–0.80) on post-ERCP day 14 (*p* < 0.001). The median absolute bilirubin reduction was 1.00 mg/dL (IQR, 0.17–5.14), corresponding to a median percentage reduction of 70.9%. Both plastic and metal stent groups demonstrated statistically significant decreases in total bilirubin levels. In the plastic stent group, median total bilirubin decreased from 1.96 mg/dL to 0.30 mg/dL (*p* < 0.001). In the metal stent group, median total bilirubin decreased from 1.19 mg/dL to 0.77 mg/dL (*p* < 0.001). Bilirubin reduction was also significant in both distal and perihilar/hilar stricture groups. The overall bilirubin response and subgroup-based bilirubin changes are presented in Table 2 and illustrated in Figure 2.

Pre-ERCP total bilirubin values showed a skewed distribution. Although the median pre-ERCP total bilirubin level was 1.50 mg/dL, the mean value was 4.74 ± 6.81 mg/dL, with a range of 0.10–41.19 mg/dL. The corresponding post-ERCP day-14 mean bilirubin level was 1.06 ± 2.28 mg/dL, with a range of 0.10–14.30 mg/dL. No pre-ERCP or day-14 bilirubin value was missing in the final cohort, and no value was imputed. The compressed day-14 distribution in the perihilar/hilar subgroup reflected the raw data distribution: 36 of 46 procedures in this subgroup had a recorded day-14 bilirubin value of 0.30 mg/dL. Nevertheless, the mean day-14 bilirubin level in this subgroup was 1.31 ± 3.03 mg/dL, with a range of 0.10–14.30 mg/dL.

Therefore, the marked median decrease in the perihilar/hilar subgroup should be interpreted as a combination of effective early biliary decompression and clustering of recorded day-14 laboratory values at the low-normal range, rather than as evidence that proximal strictures biologically respond better than distal strictures. This interpretation is particularly important because the perihilar/hilar subgroup was smaller and had a compressed day-14 median distribution.

Median total bilirubin levels before ERCP and on post-ERCP day 14 are shown for the overall cohort and predefined subgroups. A significant bilirubin reduction was observed in the overall cohort, plastic stent group, metal stent group, distal stricture group, and perihilar/hilar stricture group. ERCP, endoscopic retrograde cholangiopancreatography.

### 3.3. Clinical Outcomes and Adverse Events

Clinical drainage success was achieved in 153 of 164 procedures (93.3%). ERCP-related adverse events occurred in 18 procedures (11.0%). The most frequent adverse event was post-ERCP pancreatitis, observed in 10 procedures (6.1%), followed by cholangitis in 4 procedures (2.4%), bleeding in 3 procedures (1.8%), and perforation in 1 procedure (0.6%). Stent dysfunction occurred in 23 procedures (14.0%), and repeat ERCP was required in 23 procedures (14.0%). Among procedures with stent dysfunction, the median time to dysfunction was 28.0 days (IQR, 8.0–80.5). Thirty-day mortality occurred in 2 of 164 procedures (1.2%), consistent with Table 3. Clinical outcomes and adverse events are summarized in Table 3.

### 3.4. Comparison of Plastic and Metal Stent Groups

Compared with the plastic stent group, the metal stent group had a higher rate of definite malignancy (100.0% vs. 60.6%, *p* < 0.001), more frequent distal stricture localization (94.5% vs. 60.6%, *p* < 0.001), and larger common bile duct diameter (20.0 mm vs. 11.0 mm, *p* < 0.001). Therefore, the two stent groups were not clinically balanced at baseline. Clinical drainage success was numerically higher in the metal stent group than in the plastic stent group, but the difference did not reach statistical significance (98.2% vs. 90.8%, *p* = 0.101). Overall ERCP-related adverse events were less frequent in the metal stent group (1.8% vs. 15.6%, *p* = 0.007), and post-ERCP pancreatitis was observed only in the plastic stent group (9.2% vs. 0.0%, *p* = 0.032). Stent dysfunction and repeat ERCP rates did not differ significantly between the two groups. Detailed comparisons between plastic and metal stent groups are shown in Table 4.

Because the plastic and metal stent groups were clinically imbalanced and follow-up duration differed significantly, comparisons between stent types were interpreted descriptively rather than as evidence of comparative stent effectiveness.

### 3.5. Comparison of Distal and Perihilar/Hilar Strictures

Because distal and proximal biliary strictures may differ in anatomical complexity and drainage strategy, an exploratory subgroup analysis was performed by comparing distal strictures with combined perihilar/hilar strictures. Distal strictures accounted for 118 procedures (72.0%), whereas perihilar/hilar strictures accounted for 46 procedures (28.0%).

Metal stents were more frequently used in distal strictures than in perihilar/hilar strictures (44.1% vs. 6.5%, *p* < 0.001), while plastic stents were predominantly used in perihilar/hilar strictures.

Clinical drainage success was similar between distal and perihilar/hilar strictures (92.4% vs. 95.7%, *p* = 0.729). Stent dysfunction and repeat ERCP were numerically more frequent in distal strictures, but the differences were not statistically significant (16.1% vs. 8.7%, *p* = 0.317 for both outcomes). Because only four stent dysfunction events occurred in the perihilar/hilar subgroup, subgroup-specific multivariable models were not performed.

In univariable logistic regression, distal stricture localization was not significantly associated with stent dysfunction (OR: 2.02; 95% CI: 0.65–6.28; *p* = 0.227). These findings suggest that stricture localization influenced stent selection in real-world practice, but was not independently associated with stent dysfunction in this cohort (Table 5).

**Table 5:** Comparison of distal and perihilar/hilar biliary strictures.

Characteristic or Outcome	Distal Stricture (n = 118)	Perihilar/Hilar Stricture (n = 46)	*p* Value
Baseline characteristics			
Age, years	60.0 (44.2–71.0)	58.0 (41.5–69.0)	0.525
Male sex	51 (43.2)	26 (56.5)	0.125
ASA physical status ≥3	81 (68.6)	23 (50.0)	0.026
Definite malignancy documented	118 (100.0)	3 (6.5)	<0.001
Stent and procedural characteristics			
Plastic stent	66 (55.9)	43 (93.5)	<0.001
Metal stent	52 (44.1)	3 (6.5)	<0.001
Number of stents	1.0 (1.0–1.0)	1.0 (1.0–1.0)	0.005
Common bile duct diameter, mm	15.0 (10.0–20.0)	11.0 (10.0–15.0)	0.004
Sphincterotomy performed	115 (97.5)	45 (97.8)	1.000
Pre-cut sphincterotomy	2 (1.7)	1 (2.2)	1.000
Bilirubin response			
Pre-ERCP total bilirubin, mg/dL	1.42 (0.80–5.62)	2.75 (0.94–7.16)	0.168
Day-14 total bilirubin, mg/dL	0.57 (0.31–0.84)	0.30 (0.30–0.30)	<0.001
Clinical outcomes			
Clinical drainage success	109 (92.4)	44 (95.7)	0.729
Any ERCP-related adverse event	18 (15.3)	0 (0.0)	0.004
Post-ERCP pancreatitis	10 (8.5)	0 (0.0)	0.063
Bleeding	3 (2.5)	0 (0.0)	0.560
Perforation	1 (0.8)	0 (0.0)	1.000
Cholangitis	4 (3.4)	0 (0.0)	0.577
ICU requirement	4 (3.4)	0 (0.0)	0.577
30-day mortality	2 (1.7)	0 (0.0)	1.000
Stent dysfunction	19 (16.1)	4 (8.7)	0.317
Repeat ERCP	19 (16.1)	4 (8.7)	0.317
Time to stent dysfunction, days	76.0 (5.5–80.5)	28.0 (23.0–42.5)	0.652
Length of hospital stay, days	4.0 (3.0–5.0)	4.0 (3.0–5.0)	0.772
Follow-up duration, days	92.5 (62.0–136.2)	74.5 (62.0–93.8)	0.105

Values are presented as median (interquartile range) or n (%), unless otherwise indicated. *p* values were calculated using the Mann–Whitney U test, chi-square test, or Fisher’s exact test, as appropriate. Perihilar and hilar strictures were combined for subgroup analysis because of the limited number of proximal stricture cases.

### 3.6. Factors Associated with Stent Dysfunction

In univariable logistic regression analysis, ASA score ≥ 3 was significantly associated with stent dysfunction (OR: 4.52; 95% CI: 1.28–15.94; *p* = 0.019). Age, sex, stent type, definite malignancy, stricture localization, baseline bilirubin level, and common bile duct diameter were not significantly associated with stent dysfunction. In the restricted adjusted model including ASA score ≥ 3 and stent type, ASA score ≥ 3 remained associated with stent dysfunction (adjusted OR: 4.84; 95% CI: 1.36–17.23; *p* = 0.015). Metal stent use showed a numerical association with stent dysfunction but did not reach statistical significance (adjusted OR: 2.23; 95% CI: 0.89–5.60; *p* = 0.086). Logistic regression results are presented in Table 6.

**Figure 3:**
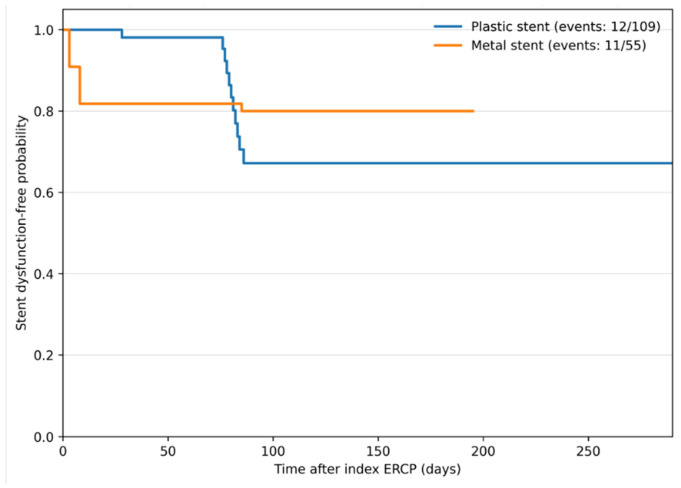
Kaplan–Meier curve for stent dysfunction-free probability according to stent type.

Kaplan–Meier analysis was additionally performed to evaluate stent dysfunction-free probability according to stent type. The log-rank test did not show a significant difference between plastic and metal stent groups in stent dysfunction-free probability during follow-up (*p* = 0.905) (Figure 3).

Stent dysfunction-free probability was estimated from the index ERCP date. Patients without documented stent dysfunction were censored at the last follow-up. Death or absence of further biliary follow-up without documented stent dysfunction was treated as censoring. The log-rank test did not show a significant difference between plastic and metal stent groups (*p* = 0.905). ERCP, endoscopic retrograde cholangiopancreatography. Because follow-up duration differed between stent groups, this Kaplan–Meier analysis should be interpreted as exploratory and descriptive. ERCP, endoscopic retrograde cholangiopancreatography.

## 4. Discussion

In this single-center real-world cohort, ERCP-guided biliary stenting in patients with suspected malignant biliary strictures was associated with high clinical drainage success and a significant early biochemical response. Among 996 analyzable ERCP records screened during the study period, 164 ERCP-guided biliary stenting procedures performed in 162 patients constituted the final study cohort. Clinical drainage success was achieved in 93.3% of procedures, and total bilirubin levels decreased significantly by post-ERCP day 14. ERCP-related adverse events occurred in 11.0% of procedures, while stent dysfunction and repeat ERCP were each observed in 14.0% of procedures. In the restricted adjusted model, ASA physical status ≥3 showed an exploratory association with stent dysfunction. These findings suggest that ERCP-guided biliary stenting is an effective early drainage strategy in suspected malignant biliary strictures, while baseline clinical condition may be relevant to subsequent stent-related outcomes.

The management of suspected malignant biliary strictures requires both diagnostic and therapeutic decision-making. In clinical practice, malignancy may be suspected at the time of ERCP but not yet confirmed pathologically, particularly when tissue sampling is limited, nondiagnostic, or deferred because urgent biliary drainage is required. Conventional ERCP-based brush cytology and intraductal biopsy are commonly used diagnostic approaches, but their sensitivity for detecting malignant biliary strictures is limited [17]. More advanced techniques, such as digital single-operator cholangioscopy with targeted biopsy, may improve diagnostic assessment in indeterminate biliary strictures, although their routine use depends on availability and local expertise [18]. Therefore, inclusion of patients with suspected malignant biliary strictures rather than only pathologically confirmed malignancy reflects an important real-world scenario in which therapeutic drainage and diagnostic clarification often proceed in parallel.

The proportion of procedures without documented definite malignancy also deserves attention. In the present cohort, 43 procedures (26.2%) were classified as having no definite malignancy documented during follow-up. This should not be interpreted as inappropriate inclusion, because the study population was defined by suspected malignant biliary stricture at the time of ERCP rather than confirmed cancer. In real-world practice, benign inflammatory strictures, stone-related obstruction with associated narrowing, postoperative or anastomotic strictures, and indeterminate strictures may mimic malignant biliary obstruction during the initial evaluation. Moreover, negative or unavailable tissue sampling does not always completely exclude malignancy. Therefore, this subgroup reflects the diagnostic uncertainty inherent to suspected malignant biliary strictures and supports the clinical relevance of studying patients at the point of endoscopic decision-making.

The high clinical drainage success rate observed in the present study supports the continued role of ERCP-guided transpapillary stenting as a central drainage approach in suspected malignant biliary obstruction. Current clinical guidance emphasizes that biliary drainage should be individualized according to stricture location, clinical presentation, expected treatment pathway, and available expertise [3,7,8]. In our cohort, the significant reduction in total bilirubin by post-ERCP day 14 provides an objective biochemical correlate of effective early decompression. This early bilirubin response is clinically relevant because adequate biliary drainage may support stabilization of patients with obstructive jaundice and facilitate further diagnostic, oncologic, or surgical planning when appropriate [8,9,10]. However, bilirubin reduction should not be interpreted as a stand-alone marker of long-term success, because subsequent outcomes may be influenced by stent patency, recurrent obstruction, cholangitis, tumor biology, and the overall oncologic pathway. The relatively high proportion of non-jaundiced procedures in this cohort reflects the real-world inclusion of patients with clinically relevant strictures identified before the development of overt hyperbilirubinemia, or in whom biliary drainage was needed to support diagnostic clarification, prevent or manage cholangitis, relieve radiological obstruction, or allow timely oncological or surgical planning. Accordingly, clinical success in these patients should be interpreted as successful clinically indicated drainage and stabilization rather than as bilirubin normalization alone.

The pronounced median bilirubin reduction observed in the perihilar/hilar subgroup requires cautious interpretation. Clinically, effective drainage of obstructed proximal biliary segments may result in rapid biochemical improvement when adequate drainage of the intended liver sector is achieved. Statistically, however, the day-14 bilirubin distribution in this subgroup was compressed, with most recorded values clustering at the low-normal range. Therefore, this finding should be interpreted as evidence of effective early decompression in successfully stented proximal strictures, rather than as a biological superiority of drainage response in perihilar/hilar strictures compared with distal strictures. The smaller size of the perihilar/hilar subgroup further supports a cautious interpretation.

Plastic and metal stents were both used in this cohort, but the two groups were clearly imbalanced at baseline. Metal stents were more frequently used in patients with definite malignancy and distal strictures, whereas plastic stents were used in a broader and more heterogeneous group, including patients without definitive malignancy at the time of analysis. Previous meta-analyses have reported that self-expandable metal stents may provide longer patency and reduce reintervention compared with plastic stents in malignant biliary obstruction [19,20]. In the present study, overall ERCP-related adverse events and post-ERCP pancreatitis were less frequent in the metal stent group; however, stent dysfunction and repeat ERCP rates did not differ significantly between plastic and metal stents. These findings should be interpreted cautiously, because stent choice was not randomized and was probably influenced by diagnostic certainty, stricture localization, expected disease course, and procedural decision-making. Therefore, our stent-type findings should be considered descriptive real-world observations rather than direct evidence of superiority of one stent type over another.

The apparent differences between plastic and metal stent groups should not be interpreted as a direct comparative advantage of one stent type, because stent selection was not randomized and was strongly influenced by baseline clinical factors. In real-world practice, metal stents were preferentially used in patients with definite malignancy, distal strictures, anticipated longer drainage requirement, and palliation or continuation of oncological treatment. In contrast, plastic stents were generally favored when the diagnosis remained indeterminate, when benign or stone-related obstruction was still possible, when temporary or removable drainage was desired, or when drainage strategy required reassessment after diagnostic clarification. For perihilar and hilar strictures, stent selection should remain individualized according to the level of obstruction, drainage target, expected treatment pathway, need for bilateral or sectoral drainage, and local expertise. Therefore, the present findings support a pragmatic decision-making framework rather than a prescriptive guideline for stent selection.

The newly performed subgroup analysis also showed that distal and perihilar/hilar strictures differed mainly in real-world management patterns rather than in clinical drainage success. Metal stents were used predominantly in distal strictures, whereas plastic stents were more common in perihilar/hilar strictures. This pattern likely reflects differences in diagnostic certainty, anatomical complexity, drainage targets, and the need for temporary versus longer-term drainage. Although stent dysfunction and repeat ERCP were numerically more frequent in distal strictures, these differences were not statistically significant, and stricture localization was not significantly associated with stent dysfunction in univariable logistic regression. Because only four stent dysfunction events occurred in the perihilar/hilar subgroup, these findings should be interpreted as exploratory.

To further account for the time-dependent nature of stent dysfunction, Kaplan–Meier analysis was performed. This analysis did not demonstrate a significant difference in stent dysfunction-free probability between plastic and metal stent groups. However, given the baseline imbalance between groups and the limited number of stent dysfunction events, this result should also be interpreted as exploratory.

A notable finding of this study was the association between ASA score ≥ 3 and stent dysfunction. This exploratory association was observed after adjustment for stent type in a restricted model. Because the number of stent dysfunction events was limited, this result should be interpreted as hypothesis-generating rather than definitive. Nevertheless, ASA score may serve as a practical indicator of baseline clinical status in patients undergoing ERCP-guided biliary stenting. Patients with higher ASA scores may have greater comorbidity burden, reduced physiological reserve, more complex peri-procedural management, and a higher likelihood of clinical deterioration or recurrent biliary events during follow-up. Although ASA score does not directly explain the mechanical mechanism of stent dysfunction, it may help identify patients who require closer post-procedural monitoring.

The overall ERCP-related adverse event profile in this cohort was clinically important but limited in frequency. Post-ERCP pancreatitis was the most frequent adverse event, followed by cholangitis, bleeding, and perforation. This spectrum is consistent with the main adverse events described in ERCP-related consensus documents and guideline-based terminology [13,14,15,16]. In our cohort, 30-day mortality was low, but this finding should not be interpreted as an oncologic survival outcome. Short-term mortality after biliary drainage in suspected malignant obstruction may be influenced by baseline clinical status, severity of obstruction, infection, tumor stage, and subsequent treatment pathway. Because only two deaths occurred within 30 days, mortality-related risk modeling was not appropriate in the present study.

The role of biliary drainage as a bridge to surgery or systemic treatment remains clinically nuanced. In patients with pancreatic head cancer, routine preoperative biliary drainage before surgery has been associated with increased complications in a randomized trial, supporting a selective rather than universal approach [21]. However, this does not eliminate the need for biliary drainage in patients with cholangitis, severe jaundice, delayed surgery, planned neoadjuvant or systemic treatment, or unresectable disease. The present cohort included a heterogeneous real-world population with suspected malignant biliary strictures, and the indication for ERCP-guided stenting was based on clinical need rather than a uniform oncologic protocol. Therefore, these results should be interpreted as outcomes of therapeutic biliary decompression in routine practice, not as evidence supporting routine drainage in all patients with pancreatobiliary malignancy.

Although ERCP-guided transpapillary drainage remains widely used, alternative biliary drainage techniques are increasingly relevant when ERCP fails or is not feasible. Endoscopic ultrasound-guided biliary drainage and percutaneous transhepatic biliary drainage have been evaluated as rescue strategies after failed ERCP [22]. In addition, randomized data have compared EUS-guided and ERCP-guided biliary drainage in malignant biliary obstruction [23]. In the present study, cannulation success was high, and the study was not designed to compare ERCP with alternative drainage modalities. Accordingly, our findings specifically address the effectiveness and safety profile of ERCP-guided biliary stenting within a real-world endoscopy unit rather than the comparative performance of different drainage routes.

Another important consideration is the duration of follow-up. Although the initial study framework aimed to evaluate early and short-term, the median follow-up duration in the final cohort was 76 days. Therefore, the present findings are more appropriately interpreted as early and short-term outcomes rather than true mid-term or long-term stent patency results. This relatively short endoscopic follow-up reflects the real-world nature of the cohort, in which patients with suspected or confirmed malignancy may be transferred to oncological care, undergo surgery, receive systemic treatment, deteriorate clinically, or continue follow-up outside the endoscopy unit. For this reason, long-term stent patency and oncological survival outcomes were beyond the scope of the present analysis.

This study has several limitations. First, the retrospective and single-center design may limit generalizability. Second, the primary unit of analysis was the ERCP-guided biliary stenting procedure; although the number of individual patients was also reported, two patients had more than one eligible procedure during the study period. Third, stent selection was not randomized, and the plastic and metal stent groups differed substantially in baseline and procedural characteristics. Therefore, comparisons between stent types are subject to selection bias and should not be interpreted causally. Fourth, the cohort included patients with suspected malignant biliary strictures rather than only pathologically confirmed malignancy. Although this reflects real-world practice, it also introduces diagnostic heterogeneity. Fifth, the number of events for some outcomes, particularly 30-day mortality and clinical drainage failure, was low; therefore, multivariable modeling was intentionally restricted to reduce the risk of overfitting. The number of stent dysfunction events was limited (n = 23). Therefore, the adjusted model was intentionally restricted to ASA physical status ≥3 and stent type to reduce overfitting. The association between ASA physical status ≥3 and stent dysfunction should be regarded as hypothesis-generating rather than as a stable or definitive independent predictor. Sixth, some procedural variables, including cannulation time and procedure duration, were not sufficiently complete for robust analysis. Finally, long-term oncologic outcomes and survival analyses were beyond the scope of this study. Transparent reporting of patient selection, missing data, outcomes, and analytical limitations is important in observational research, as emphasized by STROBE recommendations [24].

Despite these limitations, the study has several strengths. It was derived from a large screened ERCP database and focused on a clearly defined subgroup that directly corresponds to the clinical question: suspected malignant biliary strictures treated with ERCP-guided stenting. The study evaluated clinically meaningful outcomes, including drainage success, bilirubin response, ERCP-related adverse events, stent dysfunction, repeat ERCP requirement, and 30-day mortality. In addition, the analysis reflects real-world decision-making, where suspected malignancy, stricture localization, patient condition, diagnostic certainty, and anticipated treatment pathway may all influence stent selection and follow-up. These features make the findings relevant for centers managing heterogeneous patients with suspected malignant biliary obstruction in routine endoscopic practice.

In summary, ERCP-guided biliary stenting achieved high clinical drainage success and significant early bilirubin reduction in patients with suspected malignant biliary strictures. Stent dysfunction and repeat ERCP were observed in a minority of procedures, and ASA physical status ≥3 showed an exploratory association with stent dysfunction in a restricted adjusted model. Differences between plastic and metal stent groups should be interpreted within the context of real-world stent selection and baseline imbalance. Further prospective, multicenter studies with standardized definitions, predefined stent selection criteria, and longer follow-up are needed to better define predictors of stent dysfunction and optimize individualized drainage strategies in this patient population.

## 5. Conclusions

In this single-center real-world cohort, ERCP-guided biliary stenting was associated with high clinical drainage success and significant early bilirubin reduction in patients with suspected malignant biliary strictures during early and short-term follow-up.

ASA physical status ≥3 showed an exploratory association with stent dysfunction in a restricted adjusted model. However, this finding should be interpreted cautiously because of the limited number of stent dysfunction events. Although differences were observed between plastic and metal stent groups, these comparisons should be considered descriptive because stent selection was not randomized and the groups differed substantially in baseline characteristics.

Prospective multicenter studies with standardized definitions, predefined stent selection criteria, and longer follow-up are needed to better clarify predictors of stent dysfunction, long-term stent patency, and repeat intervention in this patient population.

Distal and perihilar/hilar subgroup analyses suggested that stricture localization affected real-world stent selection, but was not significantly associated with clinical drainage success or stent dysfunction in this cohort.

## Figures and Tables

**Table 1:** Baseline and procedural characteristics of the study cohort.

Characteristic	Overall Cohort (*n* = 164)
Demographic and clinical characteristics	
Age, years	60.0 (42.5–70.3)
Female	87 (53.0)
Male	77 (47.0)
ASA physical status 1	1 (0.6)
ASA physical status 2	59 (36.0)
ASA physical status 3	103 (62.8)
ASA physical status 4	1 (0.6)
ASA physical status ≥3	104 (63.4)
Malignancy status	
Definite malignancy documented	121 (73.8)
No definite malignancy documented	43 (26.2)
Stricture and stent characteristics	
Distal stricture	118 (72.0)
Perihilar stricture	25 (15.2)
Hilar stricture	21 (12.8)
Plastic stent	109 (66.5)
Metal stent	55 (33.5)
Procedural characteristics	
Common bile duct diameter, mm	13.0 (10.0–20.0)
Number of stents	1.0 (1.0–1.0)
Sphincterotomy performed	160 (97.6)
Pre-cut sphincterotomy	3/163 (1.8)
Successful biliary stent deployment	164 (100.0)
Documented cannulation status available	163/164 (99.4)
Cannulation recorded as successful	161/163 (98.8)

Values are presented as median (interquartile range) or *n* (%), unless otherwise indicated.

**Table 2:** Total bilirubin response after ERCP-guided biliary stenting.

Group	*n*	Pre-ERCP Bilirubin (mg/dL)	Day-14 Bilirubin (mg/dL)	Absolute Reduction (mg/dL)	Relative Reduction (%)	*p* Value
Overall cohort	164	1.50 (0.80–5.93)	0.42 (0.30–0.80)	1.00 (0.17–5.14)	70.9 (25.0–91.8)	<0.001
Plastic stent	109	1.96 (0.90–6.52)	0.30 (0.30–0.50)	1.37 (0.24–5.80)	81.1 (40.0–94.0)	<0.001
Metal stent	55	1.19 (0.68–3.05)	0.77 (0.64–0.90)	0.34 (−0.04–2.57)	40.0 (−7.5–77.6)	<0.001
Distal stricture	118	1.42 (0.80–5.62)	0.57 (0.31–0.84)	0.70 (0.07–3.82)	59.6 (9.9–89.2)	<0.001
Perihilar/hilar stricture	46	2.75 (0.94–7.16)	0.30 (0.30–0.30)	2.54 (0.49–6.44)	82.1 (44.0–94.6)	<0.001

Values are presented as median (interquartile range). *p* values refer to within-group paired comparisons between pre-ERCP and post-ERCP day-14 total bilirubin levels.

**Table 3:** Clinical outcomes after ERCP-guided biliary stenting.

Outcome	Overall Cohort (n = 164)
Clinical drainage	
Clinical drainage success	153 (93.3)
Clinical drainage failure	11 (6.7)
ERCP-related adverse events	
Any ERCP-related adverse event	18 (11.0)
Post-ERCP pancreatitis	10 (6.1)
Bleeding	3 (1.8)
Perforation	1 (0.6)
Cholangitis	4 (2.4)
Post-procedural course and follow-up	
ICU requirement	4 (2.4)
30-day mortality	2 (1.2)
Stent dysfunction	23 (14.0)
Repeat ERCP	23 (14.0)
Time to stent dysfunction, days	28.0 (8.0–80.5)
Length of hospital stay, days	4.0 (3.0–5.0)
Follow-up duration, days	76.0 (62.0–113.3)

Values are presented as median (interquartile range) or *n* (%), unless otherwise indicated. Time to stent dysfunction was calculated only among cases with documented stent dysfunction.

**Table 4:** Comparison of plastic and metal stent groups.

Characteristic or Outcome	Plastic Stent (n = 109)	Metal Stent (n = 55)	*p* Value
Baseline characteristics			
Age, years	60.0 (45.0–71.0)	59.0 (36.5–69.5)	0.479
Male sex	59 (54.1)	18 (32.7)	0.010
ASA physical status ≥3	71 (65.1)	33 (60.0)	0.519
Definite malignancy documented	66 (60.6)	55 (100.0)	<0.001
Distal stricture location	66 (60.6)	52 (94.5)	<0.001
Bilirubin response			
Pre-ERCP total bilirubin, mg/dL	1.96 (0.90–6.52)	1.19 (0.68–3.05)	0.011
Day-14 total bilirubin, mg/dL	0.30 (0.30–0.50)	0.77 (0.64–0.90)	<0.001
Absolute bilirubin reduction, mg/dL	1.37 (0.24–5.80)	0.34 (−0.04–2.57)	0.005
Relative bilirubin reduction, %	81.1 (40.0–94.0)	40.0 (−7.5–77.6)	<0.001
Procedural characteristics			
Common bile duct diameter, mm	11.0 (10.0–14.8)	20.0 (16.0–30.0)	<0.001
Number of stents	1.0 (1.0–1.0)	1.0 (1.0–2.0)	<0.001
Clinical outcomes			
Clinical drainage success	99 (90.8)	54 (98.2)	0.101
Any ERCP-related adverse event	17 (15.6)	1 (1.8)	0.007
Post-ERCP pancreatitis	10 (9.2)	0 (0.0)	0.032
Bleeding	3 (2.8)	0 (0.0)	0.551
Cholangitis	4 (3.7)	0 (0.0)	0.302
Stent dysfunction	12 (11.0)	11 (20.0)	0.117
Repeat ERCP	12 (11.0)	11 (20.0)	0.117
30-day mortality	1 (0.9)	1 (1.8)	1.000
Length of hospital stay, days	4.0 (3.0–5.0)	3.0 (3.0–4.0)	0.013
Follow-up duration, days	66.0 (46.0–76.0)	115.0 (100.5–154.5)	<0.001

Values are presented as median (interquartile range) or n (%), unless otherwise indicated. *p* values were calculated using the Mann–Whitney U test, chi-square test, or Fisher’s exact test, as appropriate. Because stent selection was not randomized and the groups were clinically imbalanced, these comparisons should be interpreted descriptively.

**Table 6:** Logistic regression analysis for stent dysfunction.

Variable	Univariable OR (95% CI)	*p* Value	Adjusted OR (95% CI)	*p* Value
Age, per year	1.02 (0.99–1.05)	0.171	—	—
Male sex	0.69 (0.28–1.70)	0.419	—	—
ASA physical status ≥3	4.52 (1.28–15.94)	0.019	4.84 (1.36–17.23)	0.015
Metal stent	2.02 (0.83–4.93)	0.122	2.23 (0.89–5.60)	0.086
Definite malignancy documented	2.64 (0.74–9.38)	0.133	—	—
Distal stricture location	2.02 (0.65–6.28)	0.227	—	—
Pre-ERCP total bilirubin, per mg/dL	0.98 (0.91–1.06)	0.649	—	—
Common bile duct diameter, per mm	1.01 (0.96–1.07)	0.609	—	—

The adjusted model included ASA physical status ≥3 and stent type only, owing to the limited number of stent dysfunction events. OR: odds ratio; CI: confidence interval.

## Data Availability

The data supporting the findings of this study are available from the corresponding author upon reasonable request.

## Abbreviations

ASA	American Society of Anesthesiologists
CI	Confidence interval
ERCP	Endoscopic retrograde cholangiopancreatography
EUS	Endoscopic ultrasound
ICU	Intensive care unit
IQR	Interquartile range
OR	Odds ratio
STROBE	Strengthening the Reporting of Observational Studies in Epidemiology
